# Evaluating Large Language Model–Generated Clinical Summaries Through a Dual-Perspective Framework: Retrospective Observational Study

**DOI:** 10.2196/85221

**Published:** 2026-02-10

**Authors:** Brian Han, Traci Barnes, Charitha D Reddy, Andrew Y Shin

**Affiliations:** 1 Division of Cardiology Lucile Packard Children’s Hospital Stanford University School of Medicine Palo Alto, CA United States

**Keywords:** large language models, artificial intelligence, pediatric cardiology, clinical informatics, patient advocacy

## Abstract

Large language models (LLMs) are increasingly used by patients and families to interpret complex medical documentation, yet most evaluations focus only on clinician-judged accuracy. In this study, 50 pediatric cardiac intensive care unit notes were summarized using GPT-4o mini and reviewed by both physicians and parents, who rated readability, clinical fidelity, and helpfulness. There were important discrepancies between parents and clinicians in the realm of helpfulness, along with important insights by clinicians assessing clinical accuracy and parents assessing readability. This study highlights the need for dual-perspective frameworks that balance clinical precision with patient understanding.

## Introduction

The integration of large language models (LLMs) into clinical medicine [1,2] has prompted studies to evaluate their utility in synthesizing clinical information [3], assisting with clinical decision-making [4], or answering standardized questions [5]. However, only focusing on assessments of medical fidelity may not allow appropriate assessments of optimal utility, particularly in one use case: interpretation of medical documentation for patients and their families. While patients are increasingly using LLMs to interpret medical information, systematic assessments of this use remain rare.

This gap is consequential in pediatric cardiology, where there is a layer of care complexity with involvement of a caregiver. In this setting, parents and caretakers use LLMs for improved understanding, but clarity and reassurance matter as much as clinical precision. We evaluated LLM-generated summaries of progress notes from two perspectives, clinicians and parents, introducing a 360° framework that captures complementary dimensions of utility.

## Methods

### Overview

We identified 50 patients admitted to the pediatric cardiovascular intensive care unit between July 5, 2024, and July 5, 2025. For each case, two consecutive daily progress notes were selected. Assessment and plan sections, which included relevant clinical data, were deidentified and used as input for a standardized prompt requesting a 6-to-8-sentence summary at a 6th-to-8th-grade reading level (Multimedia Appendix 1). Outputs were generated using a secure institutional version of GPT-4o mini during July 2025. Records of the children of the parent volunteers were not used as part of the study.

The generated summaries were divided among 8 pediatric cardiologists and 10 parents of pediatric cardiology patients; 2 cardiologists and 2 parents reviewed each LLM-generated summary alongside the deidentified note for reference. Parents were recruited from a local parental advocacy group and from the inpatient cardiology unit during the week of July 7, 2025. Using a 4-point Likert scale, for each summary, clinical reviewers rated clinical fidelity (accuracy, omission of information, need for revision, and clinical alignment) and helpfulness, while parental reviewers rated readability and helpfulness with separate grading rubrics (Multimedia Appendix 2). Demographic data and baseline attitudes toward LLMs were also collected.

### Ethical Considerations

The study was reviewed and approved by the institutional review board (protocol 80502). Informed consent was obtained and no compensation was provided for participation. All identifying information was omitted from patient notes and every effort was taken to preserve privacy, confidentiality, and anonymization throughout the study.

## Results

All participants completed the survey. Demographics and baseline attitudes regarding LLMs are reported in Multimedia Appendix 3. Of note, none of the parents had medical backgrounds. The composite Flesch-Kincaid grade level for the responses was 10.6. Interrater reliability (Krippendorff α) was moderate for physician grading (α=0.69) and parental grading (α=0.75). Parents reported greater familiarity and comfort with LLMs and had a stronger belief in their role in medicine than physicians. Parents consistently rated the summaries as clear, easy to understand, and helpful in explaining clinical changes. The 3 questions on helpfulness answered by parents had a Cronbach α of 0.96; the Mann-Whitney *U* test was used to compare the parents’ average scores and the physicians’ scores for 1 question. Physicians rated the summaries lower than the parents, with a significant difference (*U*=3897; *z*=2.69; *P*=.007). Separately, physicians judged clinical accuracy less favorably than parents (Table 1).

**Table 1:** Ratings of helpfulness, readability, and clinical fidelity of large language model–generated summaries for parents and physicians. All scores ranged from 1 to 4.

Questions and ratings			Scores, mean (SE)
**Perceived helpfulness by parents^a^**			
	How helpful was the summary in understanding the changes in the patient’s condition or treatment plan?	3.25 (0.58)	
	How helpful would it be to receive this summary while your child was admitted?	3.26 (0.6)	
	How helpful would this summary be in addition to the current communication you receive from the medical team?	3.36 (0.62)	
**Perceived helpfulness by physicians^a^**			
	How helpful would this summary of changes be for a patient’s family?	2.97 (0.57)	
**Parent rating of readability**			
	Readability^b^	3.36 (0.75)	
**Physician ratings of clinical fidelity**			
	Clinical accuracy^c^	3.19 (0.68)	
	Clinical completeness^d^	3.04 (0.72)	
	No need for revision^e^	2.96 (0.75)	
	Clinical alignment^f^	2.9 (0.66)	

^a^Answers ranged from “not helpful” to “extremely helpful.”

^b^Answers ranged from “hard to read” to “easy to read.”

^c^Answers ranged from “inaccurate” to “very accurate.”

^d^Answers ranged from “omitting key information” to “includes all key information.”

^e^Answers ranged from “extensive revision needed” to “no revision needed.”

^f^Answers ranged from “not aligned” to “very aligned.”

## Discussion

This study introduces a dual-perspective evaluation of LLM-generated medical summaries. While families gave favorable ratings for helpfulness and readability, there were fewer positive scores for clinical fidelity from the clinical experts. Readability scores were favorable despite the Flesch-Kincaid grade level being higher than 6 to 8, as asked for in the prompt. While the physicians still rated the summaries as helpful, their ratings were lower than those of the parents. These findings suggest that when the focus of such an assessment does not include patient and parental input, the actual patient-centered value of such summaries may be underestimated.

The discrepancies are important to understand. Patients and caregivers are using LLMs, yet validation efforts remain clinician-centric and technical [6]. Without evaluation frameworks that incorporate patient perspectives, there is a risk of limiting the potential usefulness of LLMs and our understanding of them as a patient tool [7]. For example, there were summaries that clinicians rated as having low helpfulness but that parents perceived as very helpful. It is important for physicians to acknowledge that the use of LLMs continues to grow and that laypersons have a generally positive perception of the technology [8].

Our study has several strengths and weaknesses. It used a single-institution design and a subspecialized patient population, limiting generalizability; nevertheless, it used unaltered clinical notes, enhancing real-world validity compared with curated data. It should also be noted that studies similar to this one are limited in the pediatric population, increasing the significance of this study’s impact. There was potential clustering bias in the survey distribution that was not accounted for in the statistical analyses. Another limitation was that parents reviewed summaries of notes for other children, which removed the emotional connection when evaluating information. Lastly, there was only moderate consensus among raters, which may affect the strength of the conclusions.

It is also important to acknowledge limitations related to LLM performance. For one, the Flesch-Kincaid grade level of the summaries was much higher than what the prompt dictated, indicating limitations to the simplification of complex medical information. This also limits the impact of the favorable readability ratings, as the findings may not generalize to populations with lower health literacy. Additionally, the prompt mandates a certain format to describe changes, which may force the LLM to hallucinate and overreport a change. While this was not seen in this intensive care unit population, the same prompt may not be generalizable to a more stable population. In addition, while the LLM had access to the medical plan, it processed a physician’s interpretation of objective data rather than raw data, potentially affecting its ultimate accuracy. Both limitations may have negatively affected the perceived clinical fidelity.

In conclusion, as patients continue to use LLMs, evaluations must evolve to integrate both clinical accuracy and patient experience. A balanced framework that incorporates both physicians and families should be considered to better guide safer and more effective adoption.

## Data Availability

The datasets generated or analyzed during this study are available from the corresponding author on reasonable request.

## Appendix

Multimedia Appendix 1 Standardized large language model prompt.

Multimedia Appendix 2 Physician and parental grading rubrics.

Multimedia Appendix 3 Demographics and baseline attitudes toward large language models among physicians and parents.

## Abbreviations

LLM: large language model
