# Supplementary material for: Evaluating Large Language Model–Generated Clinical Summaries Through a Dual-Perspective Framework: Retrospective Observational Study
Source: JMIR AI. 2026 Feb 10;5:e85221. doi: 10.2196/85221 (PMC12933168; doi:10.2196/85221)
Supplement: Multimedia Appendix 1 [file ai_v5i1e85221_app1.docx]

Our goal is to help patients and their families understand changes in their medical care plan. Please read the following two clinical progress notes for the same patient carefully. Your task is to explain and summarize the key changes or differences in the patient's condition, treatment plan, or recommendations between the first note and the second note in a way that is clear, accurate, and concise for a general adult audience, including those with low health literacy or limited medical knowledge. The focus should really be on changes between the two notes rather than plans made for later time.

Use simple, everyday language (at or below a 6th–8th grade reading level) and avoid all medical jargon. Please focus on the larger summary and not on specific minute details.

**Progress Note 1 dated *****. :**

**Progress Note 2 -dated ***** :**

Your explanation should be limited to 6-8 sentences maximum and should only present objective data found directly within the two progress notes. Please do not make any inferences or interpretations about how the clinician feels about the patient's progress or the clinical situation. Focus on clearly stating what has changed between the two notes, maintaining the key facts and clinical significance in a way that is easy for someone without medical training to understand.

Please format the response in the format of starting with “The changes made in the last 24 hours include…..”
