# Supplementary material for: Evaluating Large Language Model–Generated Clinical Summaries Through a Dual-Perspective Framework: Retrospective Observational Study
Source: JMIR AI. 2026 Feb 10;5:e85221. doi: 10.2196/85221 (PMC12933168; doi:10.2196/85221)
Supplement: Multimedia Appendix 2 [file ai_v5i1e85221_app2.docx]

**Provider Grading Criteria:**

| **Criterion** | **Description** | **Scoring Guidance** |  |  |  |  |  |  |
| --- | --- | --- | --- | --- | --- | --- | --- | --- |
| 1. Accuracy | How accurately does the LLM's summary reflect the changes in plan made between the two progress notes? | 4 - The summary is completely accurate and contains no errors. |  | 3 - The summary is mostly accurate with only minor inaccuracies. |  | 2 - The summary contains some inaccuracies. |  | 1 - The summary contains significant inaccuracies. |
| 2. Omission of Key Information | Does the LLM's summary omit any key information that a clinician would consider essential changes made to the plan? | 4 - The summary includes all key information. |  | 3 - The summary omits only minor details. |  | 2 - The summary omits some key information. |  | 1 - The summary omits significant key information. |
| 3. Need for Revision | How much revision would a clinician need to perform on the LLM's summary before sharing it with a patient's family? | 4 - No revision needed. |  | 3 - Minimal revision needed. |  | 2 - Moderate revision needed. |  | 1 - Extensive revision needed. |
| 4. Alignment with Clinician Summary | How closely does the LLM's summary align with how the clinician would objectively summarize the key differences in the progress notes within the limitations of the prompt (6-8 sentences with only objective changes)? | 4 - The summary aligns perfectly with the clinician's summary. |  | 3 - The summary aligns closely with the clinician's summary. |  | 2 - The summary aligns somewhat with the clinician's summary. |  | 1 - The summary does not align well with the clinician's summary. |
| 5. Helpfulness for Patient Families | How helpful would this summary of changes be for a patient’s family? | 4 - Extremely helpful. |  | 3 - Very helpful. |  | 2 - Somewhat helpful. |  | 1 - Not helpful. |

**Parental Grading Criteria:**

| **Criterion** | **Description** | **Scoring Guidance** |  |  |  |  |  |  |
| --- | --- | --- | --- | --- | --- | --- | --- | --- |
| 1. Helpfulness for Understanding | How helpful was the summary in understanding the changes in the patient's condition or treatment plan? | 4 - Extremely helpful in understanding the changes. |  | 3 - Very helpful in understanding the changes. |  | 2 - Somewhat helpful in understanding the changes. |  | 1 - Not at all helpful in understanding the changes. |
| 2. Helpfulness During Admission | How helpful would it be to receive this summary while your child is admitted? | 4 - Would be extremely helpful during admission. |  | 3 - Would be very helpful during admission. |  | 2 - Would be somewhat helpful during admission. |  | 1 - Would not be helpful during admission. |
| 3. Ease of Understanding | How easy was the summary to understand? | 4 - Extremely easy to understand. |  | 3 - Very easy to understand. |  | 2 - Somewhat easy to understand. |  | 1 - Not at all easy to understand. |
| 4. Value as a Communication Supplement | How helpful would this summary be in addition to the current communication you receive from the medical team? | 4 - Would be extremely helpful as a supplement. |  | 3 - Would be very helpful as a supplement. |  | 2 - Would be somewhat helpful as a supplement. |  | 1 - Would not be helpful as a supplement. |
