# Supplementary material for: Evaluating Large Language Model–Generated Clinical Summaries Through a Dual-Perspective Framework: Retrospective Observational Study
Source: JMIR AI. 2026 Feb 10;5:e85221. doi: 10.2196/85221 (PMC12933168; doi:10.2196/85221)
Supplement: Multimedia Appendix 3 [file ai_v5i1e85221_app3.docx]

|  | Providers (n=8) | Parents (n=10) |
| --- | --- | --- |
| Gender |  |  |
| M | 2 (25%) | 4 (40%) |
| F | 6 (75%) | 6 (60%) |
| Age |  |  |
| 20-30 years | 1 (12.5%) | 5 (50%) |
| 31-40 years | 3 (37.5%) | 4 (40%) |
| 41-50 years | 3 (37.5%) | 1 (10%) |
| >51 years | 1 (12.5%) | 0 |
| Highest Education Completed |  |  |
| College Degrees | 0 | 5 (50%) |
| Professional Degrees | 8 (100%) | 5 (50%) |
|  |  |  |
| I am comfortable with using large language models (such as ChatGPT) for various tasks. |  |  |
| Strongly Agree | 3 (37.5%) | 6 (60%) |
| Somewhat Agree | 2 (25%) | 3 (30%) |
| Neither Agree nor Disagree | 3 (37.5%) | 0 |
| Somewhat Disagree | 0 | 1 (10%) |
| Strongly Disagree | 0 | 0 |
|  |  |  |
| I have used large language models (such as ChatGPT) in the past. |  |  |
| Strongly Agree | 4 (50%) | 9 (90%) |
| Somewhat Agree | 3 (37.5) | 1 (10%) |
| Neither Agree nor Disagree | 1 (12.5%) | 0 |
| Somewhat Disagree | 0 | 0 |
| Strongly Disagree | 0 | 0 |
|  |  |  |
| I believe large language models have a place in medicine |  |  |
| Strongly Agree | 5 (62.5%) | 9 (90%) |
| Somewhat Agree | 3 (37.5%) | 1 (10%) |
| Neither Agree nor Disagree | 0 | 0 |
| Somewhat Disagree | 0 | 0 |
| Strongly Disagree | 0 | 0 |
